# Exploring changes to financial protection and equity in Lithuania following 2017–2020 policies to improve access to outpatient medicines^☆^

**DOI:** 10.1016/j.hpopen.2026.100166

**Published:** 2026-02-24

**Authors:** Liubovė Murauskienė, Marina Karanikolos

**Affiliations:** a Public Health Department, Faculty of Medicine, Vilnius University, Vilnius, Lithuania; b European Observatory on Health Systems and Policies, London School of Hygiene and Tropical Medicine, London, UK

**Keywords:** Lithuania, Access to medicines, Catastrophic health spending, Pharmaceutical policy, Financial protection

## Abstract

**Introduction:**

Access to pharmaceuticals is a key area of health systems performance. Previous analyses showed medicines to be the largest contributor to catastrophic spending in Lithuania, particularly for people with lowest incomes. This paper examines changes to household spending on health following 2017–2020 pharmaceutical policies to improve access to outpatient medicines.

**Methods:**

Household spending and catastrophic health spending were calculated using household budget survey data for 2016 and 2021. Pharmaceutical policies were obtained from the review of legislation and regulation, and publications by the relevant health authorities.

**Results:**

Catastrophic spending in Lithuania reduced from 11.5% in 2016 to 9.4% in 2021, driven by reductions in out-of-pocket spending for medicines and with significant decrease in the poorest income quintile and among people aged 75 + . The measures introduced in 2017–2020 included eliminating percentage co-payments for nearly all covered medicines, as well as exempting people aged 75 + and some other groups on low income from co-payments for medicines.

**Conclusions:**

In 2017–2020 measures were implemented reduce the burden of household spending on medicines, resulting in reduction of catastrophic spending in the most vulnerable groups. Challenges remain, as still 9% of households experience catastrophic spending. Next steps could involve broadening exemptions, addressing gaps in the positive list, and understanding accessibility barriers in other health care services.

## Highlights

• Access to medicines in Lithuania has been an ongoing priority for policy makers.
• Policies aiming to reduce co-payments were introduced in 2017–2020.
• Catastrophic spending reduced with large improvements in the poorest and older people.
• In 2021 one in 9% of households faced financial hardship due to spending on health.
• Monitoring access barriers to services is needed to improve coverage design.

## 1 Introduction

Access to pharmaceuticals is a key area of health systems performance, assessing whether people can obtain necessary medications without facing financial hardship. However, even in many high-income countries balancing pharmaceutical spending and access continues to be a persistent challenge. Thus, access to medicines varies widely not only across health systems, but also within, causing inequities and often placing damaging financial burden on households that are most vulnerable [1]. Inadequate access to necessary medicines contributes to poorer health outcomes, exacerbates health disparities, and adds to impoverishment [2].

Access to medicines is influenced by a variety of factors, with some, such as global supply chains, manufacturing processes and international agreements, being often beyond a country’s control. Others, however, relate to national contexts, such as economic constraints, political priorities, regulatory policies and health systems capacity to assess and improve effective coverage for medicines.

In Lithuania, modern era of governing pharmaceutical sector began with the introduction of the Law on Pharmacy in 2006. The issue of access to pharmaceuticals has received more focused attention as a result of the global financial crisis in 2009 and with the subsequent austerity measures aimed at reducing prices of medicines, as well as more rational prescribing and dispensing policies [3], [4]. In 2016 the State Audit report found that many reimbursed generic medicines were predominantly funded out-of-pocket, with amount of co-payments increasing over time [5]. Among the recommendations of the report there was one for the Ministry of Health to reduce financial burden on patients. However, at a time the evidence of the impact of out-of-pocket payments for pharmaceuticals on financial protection and equity was scarce. In 2018 the analysis by the WHO Barcelona Office on Health Systems Financing [6] have shown medicines to be the largest contributor to catastrophic spending in Lithuania, particularly adversely affecting people from the poorest income quintile.

Since 2017 series of measures related to reimbursement and aimed at reducing financial burden of medicines have been introduced in Lithuania. By 2025, these included exemptions from co-payments for vulnerable groups, removing percentage co-payments, consolidating positive lists of reimbursed outpatient medicines, and setting the cap on co-payments [7]. Further measures tackled medicines pricing, supply and demand. As a result, growth in out-of-pocket spending on medicines that averaged at 6% annually – from EUR PPP 157 per capita in 2006 to EUR PPP 267 per capita in 2016 – has reversed and stalled, and in 2023 out-of-pocket spending still remains at EUR PPP 237 per capita – same as in 2017 [8].

This paper uses household budget survey data to analyse household spending on health and evaluates how reforms introduced in Lithuania between 2017 and 2020 to reduce out-of-pocket spending on retail outpatient medicines affected financial protection and equity in access to medicines.

## 2 Methods

### 2.1 Analysis of financial protection and equity

Analysis of data from household budget survey was conducted in line with the methodology developed by the WHO Barcelona Office for Health Systems Financing [9], [10], [11]. There, capacity to pay is calculated as total household’s consumption minus a normative (standard) amount to cover basic needs such as food, housing and utilities (see Appendix Table A1). This amount is deducted for all households and is referred to as a poverty line or basic needs line, and threshold for catastrophic spending is set at 40% of household’s capacity to pay. Levels of household income were determined by levels of household consumption. Household expenditures are reported in current EUR per person. This methodology better accounts for the impact of out-of-pocket payments on households by using a more sensitive metric for capacity to pay for health care, in comparison to the alternative indicator set to measure universal health coverage (UHC) progress in Sustainable Development Goal (SDG) 3.8.2 globally, where a 10% threshold applies to health spending irrespectively of household’s level of income/consumption.

The level of monetary out-of-pocket spending on health-related services and the level of catastrophic spending were used as the main indicators of financial protection, disaggregated by type of care (medicines, medical products (e.g. glasses, hearing aids, orthopaedic appliances, insulin pumps), dental care, outpatient care, inpatient care and diagnostics). Equity dimension has been analysed by assessing changes to household spending by consumption quintile and age (under 45 year olds, 45–64, 65–74 and 75 + year olds). Equity here is conceptualized as an extent to which the distribution of financial protection is fair [12], implying that differences in financial protection (catastrophic spending) across income and age are inequitable.

In this study we refer to out-of-pocket payments (household expenditure on health) as any payment made by people at the time of using any health good or service provided by any type of provider [11]. These include: co-payments (user charges) – a cost-sharing element where people pay part of the cost of health good or services covered by the benefits package; direct payments – where people pay for health goods or services not covered by the benefits package; and informal payments – unofficial additional direct contribution for health goods or services to which patients are entitled.

### 2.2 Household budget survey data

To determine the levels of catastrophic health spending, analysis uses the data from the household budget survey (HBS). HBS is a nationally representative survey carried out in Lithuania periodically since 1992 and adhering to standardized EUROSTAT methodology since 2003 [13]. In Lithuanian HBS it is not possible to distinguish between different types of out-of-pocket payments (co-payments, direct payments, and informal payments); only monetary payments were included in the analysis. The HBS data were obtained from the Statistics Lithuania [14] for year 2016 (year preceding implementation of the explored reforms) and 2021 (latest available year). Sample sizes were 3443 for year 2016 and 4334 for year 2021. Additional quality checks were carried out to validate whether out-of-pocket spending on health derived by the analysis equates to that published by Statistics Lithuania. Analyses were carried out using Stata v.18.

### 2.3 Review of policies related to reducing household spending on medicines

Policies implemented in 2017–2020 were obtained from the review of national legislation and regulation related to medicines, and publications by the Ministry of Health of the Republic of Lithuania, the National Health Insurance Fund (NHIF) and the State Medicines Control Agency (SMCA). Policies were classified into pricing, reimbursement and other measures, in line with previously published literature that maps medicines policy interventions in Europe [4].

## 3 Results

### 3.1 Households’ out-of-pocket spending on health

**Fig. 1:**
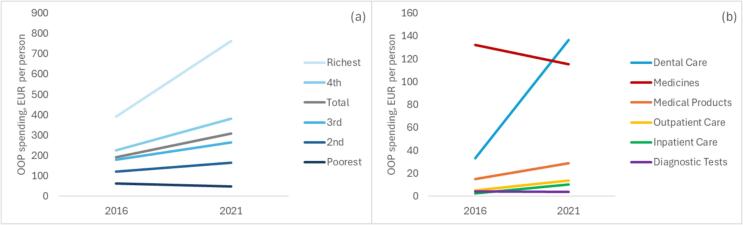
Out-of-pocket spending per person by income quintile (a) and type of care (b).

Analysis of HBS data for Lithuania shows that total out-of-pocket (OOP) spending on health reported by Lithuanian households has increased on average from EUR 193 per person in 2016 to EUR 309 in 2021. However, the changes varied across income quintiles and across types of care (Fig. 1).

The poorest income quintile saw a reduction in OOP spending (from EUR 63 to EUR 48 per person), while in all other quintiles OOP spending increased progressively with income, with relatively highest increase in the richest quintile (from EUR 392 to EUR 764 per person). For types of care, medicines were the largest driver of OOP spending in 2016, with EUR 132 per person, but reduced to EUR 115. In contrast, spending on dental care has increased markedly, from EUR 33 per person in 2016 to EUR 137 in 2021 respectively, and taking over from medicines as the largest driver. Most other types of care contributed substantially less in both years, with increase seen in medical products (from EUR 15 to EUR 29), outpatient care (from EUR 5 to EUR 14) and inpatient care (from EUR 3 to EUR 10), with slight decrease in diagnostics that still remained around EUR 4 per person in both years.

Further disaggregation by type of care in each quintile shows substantial reduction in OOP spending on medicines in the two poorest as well as the richest quintile, and very small reductions in the 3rd and 4th quintile (Appendix Fig. A3). Dental care, on the contrary, has seen a very large growth in absolute terms in all quintiles, ranging from EUR 6 per person increase in the poorest quintile, to EUR 352 in the richest quintile (similar pattern but on a smaller scale was seen in outpatient and inpatient care); OOP payments for medical products increased the least in the richest quintile, while in diagnostic tests it only increased in the 3rd and 4th quintile, and reduced in the richest.

### 3.2 Households’ catastrophic health spending

**Fig. 2:**
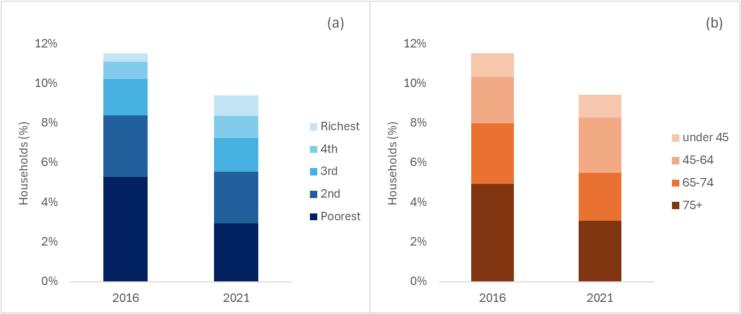
Share of households with catastrophic health spending by income quintile (a) and age group (b).

HBS analysis showed that catastrophic health spending in Lithuania reduced from 11.5% in 2016 to 9.4% in 2021 (Fig. 2). In 2016 two poorest quintiles accounted for almost three quarters of all households with catastrophic health spending, while in 2021 their share reduced to just over half. The reduction was particularly marked in the poorest quintile, where prevalence of catastrophic health spending reduced from 26% in 2016 to 15% in 2021 (Appendix Table A2). The two richer quintiles saw a small increase in absolute terms. In terms of age, catastrophic health spending predominately affected people of 65 years old in both 2016 and 2021. There has been a significant reduction in prevalence of catastrophic health spending among the 75 + year olds – from 40% in 2016 to 22% in 2021 (Appendix Table A2). At the same time, in 2021 prevalence of financial hardship reduced to a smaller extent in people aged 65–74 (from 19% to 15%), and increased in people aged 45–64 (from 6% to 8%).

**Fig. 3:**
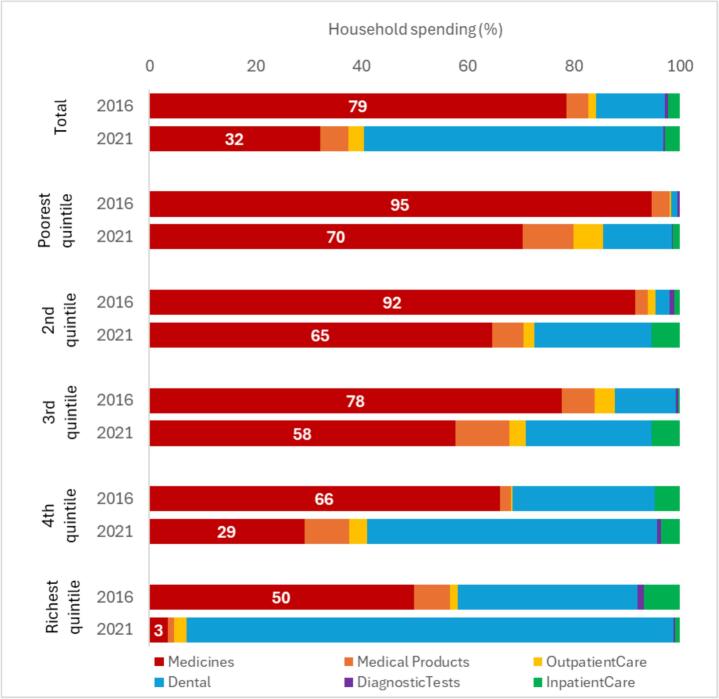
Share of catastrophic health spending in each quintile by type of care.

Further disaggregation of catastrophic health spending by type of care in each quintile shows change in the role of different types of care between 2016 and 2021 (Fig. 3). On average, the share of medicines in catastrophic health spending reduced from 79% in 2016 to 32% in 2021. In 2016 medicines were the driver of catastrophic health spending in all quintiles, but their share varied from 95% in the poorest to 50% in the richest quintile. In 2021 medicines were still dominant in the three poorest quintiles, but their share reduced to between 70% (poorest quintile) and 58% (3rd quintile). In the 4th and the richest quintiles the share of medicines has dropped markedly – to 29% and only 3% respectively. This shift largely occurred due to reduction in household spending on medicines across most quintiles (see Appendix Fig. A3), but also due to increase in spending on dental care. On average, dental care accounted for 13% of catastrophic health spending in 2016, increasing to 56% in 2021. While hardly visible in 2016 in the two poorest quintiles, it plays much larger role by 2021, ranging from 13% in the poorest quintile to 92% in the richest quintile. Other types of care play much smaller role, although it is notable that the share of medical products only increased in the three poorest quintiles.

### 3.3 Health coverage policies related to accessibility of pharmaceuticals 2017–2020

In 2016, prescribed reimbursed outpatient medicines in Lithuania were covered by compulsory health insurance according to one of the two lists (List A and List B) at a rate of 50%, 80%, 90% or 100%, depending on the diagnosis, medicine, disability level, special needs, and whether a patient was a child, working adult, or older-age pensioner. There was no cap on co-payments, and all patients had to also pay the difference between reimbursement and retail price. Prescribing by International Nonproprietary Names (INN) was formally in place and pharmacists were obliged to encourage patients to choose pharmaceutical analogue with cheapest co-payment.

Since 2017 series of measures to tackle pharmaceutical spending (both public and out-of-pocket) were introduced, with policies implemented between 2017 and 2020 relating mainly to improving coverage and affordability of reimbursed outpatient medicines (Table 1). Most measures introduced in that time frame related to reimbursement of medicines, including reviewing the criteria for reimbursement, reducing percentage co-payments, and introducing exemptions from co-payments for certain population groups. In addition, policies tackled pricing of pharmaceuticals requiring prescription, better monitoring of utilization of reimbursed pharmaceuticals, and measures to reduce demand of less cost-effective medicines.

**Table 1:** Pharmaceutical policies related to reducing out-of-pocket spending on medicines in Lithuania 2017–2020.

Policy type	Measure	Time of change / Source
Pricing Policies	Amendments to the law of value added tax (VAT) were introduced setting a reduced VAT rate of 5% on prescription non-reimbursable medicines. Previously, VAT at 5% was only applicable to prescription reimbursable medicines.	January 2018 / Parliament of the Republic of Lithuania (2002) Law on value added tax Nr. IX-751 [33]
	Grouping single-manufacturer pharmaceuticals by INN, pharmaceutical form and potency to identify supply vulnerabilities, and reviewing reimbursement price upwards.	July 2018 / Minister of Health (2000) Order on the approval of the lists of reimbursed medicines [34]
Reimbursement policies	Setting a cap for patient co-payments for more expensive medicines that have a generic analogue.	August 2017 / Ministry of Health (2017) Order of the Minister of Health on the approval of pharmaceutical policy guidelines [35]
	Review the criteria for reimbursement levels of medicines.	August 2017 Ministry of Health (2017) Order of the Minister of Health on the approval of pharmaceutical policy guidelines [35]
	The cap on paying the difference between reimbursement and retail price was set at 25% of an average reimbursed prescription price from previous year, amounting to the maximum of EUR 4.71 per package (including VAT).	July 2018 / Minister of Health (2000) Order on the approval of the lists of reimbursed medicines [34]
	Removing medicines where the co-payment for the difference between reimbursement and retail price exceeded EUR 4.71 from the list of reimbursed pharmaceuticals, except for medicines that are not substitutable (in which case reimbursement was recalculated to leave the maximum co-payment at the level of EUR 4.71.	July 2018 / Minister of Health (2000) Order on the approval of the lists of reimbursed medicines [34]
	Increasing the reimbursement rate of pharmaceuticals for treatment of CVDs and disorders of lipoprotein and other lipidemia from 80% to 90%; and increasing the reimbursement rate of all other pharmaceuticals reimbursed at 80% and 90% rate to 100%.	July 2018 / Minister of Health (2000) Order on the approval of the lists of reimbursed medicines [34]
	Reimbursement rate of pharmaceuticals for treatment of CVDs was increased from 90% to 100%.	January 2019 / Minister of Health (2000) Order on the approval of the lists of reimbursed medicines [34]
	Reimbursement rate of all pharmaceuticals which were previously reimbursed at 50% (e.g. pharmaceuticals for treatment of Herpes virus, Lyme disease, etc.) was increased to 100%	April 2019 / Minister of Health (2000) Order on the approval of the lists of reimbursed medicines [34]
	Cover co-payments from the state budget for insured people aged 75 and older and for people who are disabled or have reached retirement age and have low income (e.g. below EUR 257 per month in 2020).	July 2020 / Parliament of the Republic of Lithuania (1996) Health insurance law Nr. I-1343 (2019 amendment Nr. XIII-2492) [34]
Other measures	Implementing monitoring system to track co-payments for reimbursed medicines.	August 2017 / Ministry of Health (2017) Order of the Minister of Health on the approval of pharmaceutical policy guidelines [35]
	Developing prescribing policy that encourages physicians to prescribe cheapest pharmaceutical analogue.	August 2017 / Ministry of Health (2017) Order of the Minister of Health on the approval of pharmaceutical policy guidelines [35]
	Further enforcement of prescription by INN and obligation for pharmacists to offer a medicine with cheapest co-payment first.	July 2018 / Minister of Health (2000) Order on the approval of the lists of reimbursed medicines [36]

## 4 Discussion

The analysis of HBS data and pharmaceutical policies in Lithuania between 2016 and 2021 shows that measures aimed mainly at lowering co-payments for reimbursed pharmaceuticals were followed by reduction in household spending on retail outpatient medicines. The measures introduced in 2017–2020 included eliminating percentage co-payments for nearly all covered medicines, as well as exempting people aged 75 years and older and some other groups on low income from co-payments. The resulting reductions in OOP spending for medicines were particularly pronounced for the households in the two poorest quintiles. As a result, catastrophic health spending has reduced specifically (and significantly) among households from poorest quintile and households headed by a person aged 75 years or older, thus improving equity of access and financial protection. Nevertheless, 9% of households in Lithuania experienced catastrophic health spending in 2021, and medicines still remain the largest driver of catastrophic health spending in the three poorest quintiles.

The use of HBS is crucial to construct the indicator of catastrophic health spending that allows to assess the levels of health system’s contribution to its key goals – financial protection and equity, and progress towards UHC [2]. In this case, the analysis shows that policies implemented in Lithuania had a positive impact on reduction of catastrophic health spending that was driven by pharmaceuticals, for the poorest quintile and people over 75 years old. As many of those households are likely to already live under or close to poverty threshold, it is essential to keep them protected from further impoverishment by expanding coverage in a way that is tailored to the most vulnerable groups. In addition, HBS analysis shows that in 2021 households headed by people aged 65 and over are remain dominant in the structure of catastrophic health spending, while poorest quintiles continue to be disproportionately affected, therefore further strengthening protections from financial hardship due to use of health services remains vital. The importance of having effective protections for vulnerable groups from out-of-pocket spending on reimbursable medicines has been demonstrated in previous studies [15], [16].

Pharmaceutical accessibility is a major area of concern for policy makers across the EU and beyond [17], [18], [19]. On one hand, pharmaceutical spending takes up a substantial share of public spending on health, while on another – it remains one of the healthcare areas with the largest reliance on out-of-pocket spending across the EU [8]. In Lithuania, only 48% of pharmaceutical spending was publicly funded in 2021, compared to 59% in the EU on average [7]. Internationally harmonized national health accounts data show that in 2016 Lithuania had the second-highest out-of-pocket spending on pharmaceuticals in the EU after Bulgaria, with EUR PPP 267 (and highest level since data became available). Since early 2000s, out-of-pocket spending on pharmaceuticals decreased only three times: in 2009 (when the GDP dropped by 15% due to the financial crisis), in 2013 (when austerity policies were introduced to tackle public spending, including cost of pharmaceuticals) [3], and in 2017–2021 (with the exception of 2019) [8]. In the first two instances OOPs for pharmaceuticals had only reduced marginally, by less than EUR PPP 5. Reductions in 2017 were more substantial, reaching EUR PPP 221 in 2018 and in 2023 still remaining below the level of 2016 with EUR PPP 237. However, total OOP spending on medicines in Lithuania is still among the highest in the EU context, where average in 2023 is EUR PPP 147 [8].

At the same time, the NHIF data show that patient co-payments for reimbursed pharmaceuticals and medical aids (combined, although pharmaceuticals represent over 90% of the total) reduced from EUR 20 per person in 2016 to EUR 6.26 in 2021, before increasing again to EUR 9.08 by 2024. Co-payments covered by the state budget that was in place since mid-2020 have increased from EUR 5.59 in 2021 to EUR 10.88 in 2024, while reimbursement for medicines per person increased from EUR 79 in 2016 to EUR 139 in 2021 to EUR 206 in 2024 [20]. Policies implemented in Lithuania during that period were mainly aimed at reducing co-payments for medicines, as well as rational purchasing of medicines reimbursed by the NHIF thus tackling the demand side and maximizing the benefits across the two areas.

Pharmaceutical policy in Lithuania in 2010–2015 has mainly focused on pricing [4]. Since 2016 the focus was tailored more towards making pharmaceutical spending more rational, while expanding pharmaceutical coverage [21]. Strengthening prescribing by INN, ramping up e-prescribing, regular revision of positive list, prioritizing coverage of lower-cost analogue, simplifying levels of reimbursement and lists of reimbursed medicines, exempting older people and vulnerable people with low incomes have followed the international recommendations for strengthening financial protection and striving towards UHC have all been in line with international best practice [1].

Yet, there were also unintended consequences manifesting in particular in shortages of medicines. Much of it was related to global developments – often triggered by supply chain disruptions and manufacturing issues [18], complicating efforts to ensure consistent access to essential medicines. However, some were linked to the pharmaceutical industry’s response to policies implemented at the national level, resulting in discontinuation of supplying certain medicines to less profitable markers [22], [23], [24]. As a result, health authorities in Lithuania set up a weekly mechanism of reporting on availability of reimbursed medicines, obliging pharmacies to make a required pharmaceutical available to patient within 2–4 days (depending on geographical location), and triggering action by the Ministry of Health and the NHIF if critical shortages have been identified [25], [26]. In 2023, the National Audit Office again reviewed the issue of accessibility of pharmaceuticals and found that 30% of pharmaceuticals with authorisation are not available on the Lithuanian market [27].

Further pharmaceutical measures relating to co-payments were implemented since 2021. These include abolishing positive list B and percentage co-payments (i.e. making all medicines on a single positive list fully reimbursed) in 2023, setting an annual cap on co-payments for reimbursed medicines in 2024 (EUR 59.04 in 2025), and gradually increasing cap on co-payments for difference between reimbursement and retail price per item (from up to EUR 4.71 in 2021 to up to EUR 5.87 in 2025). The latter means an estimated 25% increase in co-payments (the data includes co-payments for both reimbursed medicines as well as medical appliances) per person over one year (from EUR 7.28 in 2023 to EUR 9.08 in 2024), although in comparison to 2016, co-payments have still halved [20].

Nevertheless, the bulk of OOP spending on pharmaceuticals does not relate to reimbursed medicines: only about 70% of prescribed retail outpatient medicines are reimbursed, leaving almost a third of medicines deemed necessary for treatment outside of the benefits package [28], in addition to substantial share spent of medicines purchased over-the-counter (OTC). This is in part due to reimbursement rules being linked to specific clinical and sometimes demographic criteria, as well as dosage and form of medication, that sometimes may leave patients paying full price for a medicine that was prescribed but could not be reimbursed due to these rules or not available in the pharmacy. Medicines prescribed by providers that are not contracting with the NHIF are not reimbursed either. At the same time, reducing OTC spending would rely on expanding coverage of prescribed medicines, enhanced regulation of pharmaceutical services as well as improving population health literacy and communication [29].

Looking at broader financial protection analysis beyond outpatient medicines, increase in OOP for dental care and the share of dental care responsible for catastrophic health spending in the richer quintiles suggests that this area also requires further policy attention. It is likely that lack of OOP spending on dental care in the poorer quintiles reflects a degree of unmet need [1].

The study has a number of limitations. First, household spending on health is derived from household budget survey and has typical limitations of this type of data source, such as potential issues with sampling and representativeness, as well as reliance of self-reporting; it is also not possible to distinguish between spending on prescribed medicines from over-the-counter medicines and food supplements. However, the HBS methodology is robust and has been harmonized across the EU countries, and is widely used by the statistical offices [30]. Comparisons with national health accounts data published by Statistics Lithuania show that the levels of household spending reported in HBS is an underestimate of total OOP spending derived from administrative data sources, with the main discrepancy being outpatient care [31]. Second, while pharmaceutical policies were implemented in years between the 2016 and 2021 HBS waves, other factors could potentially affect household spending on health, including changes in overall household income. However, reduction in household spending on medicines specifically is contrasting to increase OOP spending in most other areas of health care, suggesting the positive impact on financial protection and equity is specific to pharmaceutical policies. Finally, COVID-19 pandemic had an impact on access to services in Lithuania [32]. While it is challenging to fully account for dynamics in service provision disrupted by the pandemic, it is likely that at a time of HBS survey essential health care services were returning to routine operations. The next HBS wave is scheduled for 2026 with data expected to become available no earlier than 2027.

## 5 Conclusions

Our findings have important policy implications. Pharmaceutical spending in Lithuania has been relatively high, with the share reliant on out-of-pocket payments being one of largest in Europe. Important policies were put in place since 2016 to reduce the burden of OOP on medicines for households, resulting in reduction of catastrophic spending, particularly for the poorer households and older people. Yet, challenges for policy makers around the level of financial hardship caused by medicines in Lithuania remain, as still 9% of households report experiencing catastrophic health spending, with medicines still being the largest driver in the poorer households, although with notable shift towards other types of care. Next steps could involve broadening exemption from co-payments for medicines for people starting from 65 years old, addressing gaps in medicine coverage in the positive list, as well as exploring accessibility of other health care services, most notably outpatient care, including dental care.

## CRediT authorship contribution statement

**Liubovė Murauskienė:** Writing – review & editing, Writing – original draft, Methodology, Formal analysis, Data curation, Conceptualization. **Marina Karanikolos:** Writing – review & editing, Writing – original draft, Supervision, Methodology, Conceptualization.

## Funding

The analysis was funded by the World Health Organization (WHO) Regional Office for Europe and the European Commission (EU4Health programme) as a part of a multi-country project led by the WHO Barcelona Office for Health Systems Financing to monitor affordable access to health care (financial protection), strengthen the evidence base on universal health coverage and support policy development in health systems in Europe.

## Declaration of competing interest

The authors declare the following financial interests/personal relationships which may be considered as potential competing interests: Liubovė Murauskienė reports financial support was provided by The WHO Regional Office for Europe and the European Commission (EU4Health programme). Other authors declare that they have no known competing financial interests or personal relationships that could have appeared to influence the work reported in this paper.
