# Supplementary material for: Exploring changes to financial protection and equity in Lithuania following 2017–2020 policies to improve access to outpatient medicines
Source: Health Policy Open. 2026 Feb 24;10:100166. doi: 10.1016/j.hpopen.2026.100166 (PMC12964039; doi:10.1016/j.hpopen.2026.100166)

### Supplementary material / Appendices

**Appendix Table A1. Calculation of catastrophic health spending (WHO Barcelona Office for Health System Financing methodology)**

| **Definition** | The share of households with out-of-pocket payments that are greater than 40% of household capacity to pay for health care. This includes all households who are impoverished after out-of-pocket payments (because they no longer have any capacity to pay) and further impoverished (because they have no capacity to pay before or after out-of-pocket payments). |
| --- | --- |
| **Numerator** | Out-of-pocket payments |
| **Denominator** | A household’s capacity to pay for health care is defined as total household consumption minus a standard amount to cover basic needs; the standard amount is calculated as the average amount spent on food, housing and utilities by households between the 25th and 35th percentiles of the household consumption distribution, as described above; this standard amount is also used as a *poverty line* (basic needs line) to measure impoverishing health spending |
| **Disaggregation** | Results are disaggregated into household quintiles by consumption per person using Organisation for Economic Co-operation and Development equivalence scales; disaggregation by place of residence (urban–rural), age of the head of the household, household composition and other factors is included where relevant |
| **Data source** | Microdata from national household budget surveys |

Source: UHC Watch Glossary [11]

**Appendix Table A2. Incidence of catastrophic health spending within income quintiles and age groups**

|  | 2016 (n=3443) | 95% CI | | 2021 (n=4334) | 95% CI | |
| --- | --- | --- | --- | --- | --- | --- |
| Total share of households with catastrophic spending (%) | 11.5423 | 10.3267 | 12.7579 | 9.4175 | 8.4253 | 10.4098 |
| Incidence of catastrophic spending among income quintiles (%) |  | | | | | |
| 1st (poorest) | 26.4568 | 30.2310 | 14.7080 | 14.7080 | 12.1843 | 17.2318 |
| 2nd | 15.4972 | 18.6094 | 13.0147 | 13.0147 | 10.5681 | 15.4612 |
| 3rd | 9.1476 | 11.6725 | 8.5024 | 8.5024 | 6.5334 | 10.4713 |
| 4th | 4.4050 | 6.0576 | 5.5498 | 5.5498 | 3.8173 | 7.2823 |
| 5th (richest) | 2.1943 | 3.3637 | 5.3076 | 5.3076 | 3.0838 | 7.5315 |
| Incidence of catastrophic spending among age groups (%) |  | | | | | |
| 0-44 | 3.7202 | 2.4182 | 5.0222 | 3.3022 | 1.8213 | 4.7831 |
| 45-64 | 6.0512 | 4.7870 | 7.3154 | 7.6725 | 6.0858 | 9.2593 |
| 65-74 | 18.6490 | 15.0319 | 22.2660 | 15.2015 | 12.6043 | 17.7987 |
| 75+ | 39.8480 | 34.3644 | 45.3317 | 22.2821 | 19.1972 | 25.3670 |

**Appendix Figure A1. Out-of-pocket spending by type of care in each income quintile**


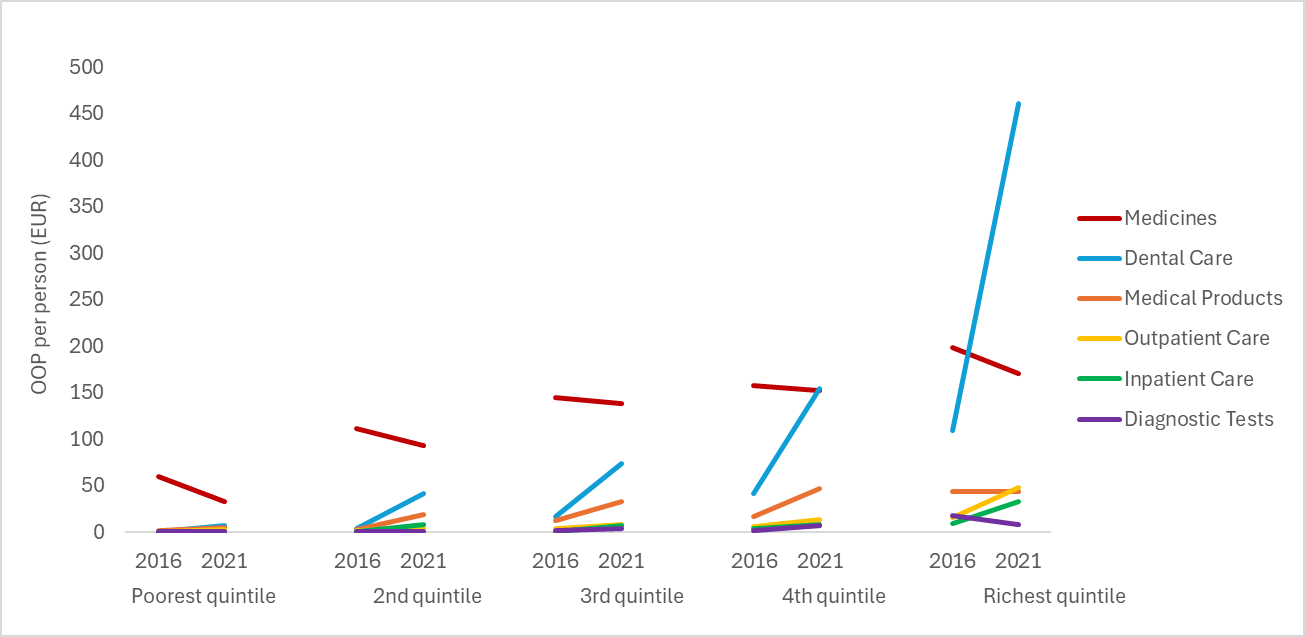

Supplement: Supplementary Data 1 [file mmc1.docx]
